# Factors Associated with Condom Knowledge, Attitude, and Use among Black Heterosexual Men in Ontario, Canada

**DOI:** 10.1155/2021/8862534

**Published:** 2021-05-03

**Authors:** Josephine Etowa, Bishwajit Ghose, Hughes Loemba, Egbe B. Etowa, Winston Husbands, Francisca Omorodion, Isaac Luginaah, Josephine Pui-Hing Wong

**Affiliations:** ^1^Faculty of Health Sciences, School of Nursing, University of Ottawa, Ottawa, Ontario, Canada; ^2^Montfort Hospital, Ottawa, Ontario, Canada; ^3^Ontario HIV Treatment Network, 1300 Yonge Street, Suite 600, Toronto, ON M4T 1X3, Canada; ^4^Department of Sociology, Anthropology & Criminology, University of Windsor, Windsor, Canada; ^5^Department of Geography, Western University, London, Ontario, Canada; ^6^Daphne Cockwell School of Nursing, Faculty of Community Services, Ryerson University, Toronto, Ontario, Canada

## Abstract

African, Caribbean, and Black (ACB) men living in Canada share a heightened risk of HIV infection and the associated risk factors such as suboptimal use of family planning services such as condom use. In this study, we assessed the factors associated with knowledge, attitude, and condom use among ACB men in Ontario. *Methods*. This was a cross-sectional study that surveyed heterosexual ACB men regardless of their residency status living in Ontario (*n* = 430). This is a part of a larger mixed methods study informed by critical race theory, intersectionality, and community-based participatory research (CBPR). Outcome variables were knowledge of condom use, attitude towards condom use, and actual use of condom during the last 12 months. *Results*. Of 430 participants, 77.70% has good knowledge of condom use as a protection against HIV transmission, 31.77% had positive attitude towards condom use, and 62.43% reported using condom regularly with casual partners during the last 12 months. Men who were currently married had more positive attitude towards condom use compared with their unmarried counterparts (odds ratio = 1.46, 95% CI = 1.20, 1.78). Canadian residents were found to have higher odds of having correct knowledge of condom (odds ratio = 1.31, 95% CI = 1.11, 1.55), and positive attitude towards condom use (odds ratio = 1.44, 95%CI = 1.09, 1.92). Men who visited sexual health clinics showed a positive association with having correct knowledge of condom (odds ratio = 1.78, 95% CI = 1.30, 2.44) and reported experiences of difficulty in accessing sexual health. This showed a negative association towards condom use (odds ratio = 0.45, 95% CI = 0.21, 0.97]. *Conclusion*. A considerable percentage of heterosexual ACB men did not have correct knowledge regarding the protective effect of condom use against HIV and positive attitude towards the use of condom. Several sociodemographic and healthcare-related factors were significantly associated with knowledge, attitude, and use of condom.

## 1. Introduction

Globally, condom use is an important method of family planning and prevention of sexually transmitted infections, especially HIV/AIDS in order to improve health and quality of life [2]. Universal provision and utilization of family planning (FP) services is regarded as one of the key strategies to achieving several sustainable development goals (SDGs), especially those relevant to prevention of life-threatening diseases such as HIV/AIDS [3–5]. A great majority of the population are aware of some form of modern contraceptive methods and have reported willingness to adopt a method of birth control and family planning [6, 7]; nonetheless, unmet need for and nonuse of FP are still widely prevalent and is leading to high rates of HIV infections among marginalised population such as the African, Caribbean, and black communities [4, 8–11]. Studies have shown that when used consistently and correctly, condoms are highly effective in preventing the transmission of a range of sexually transmitted infections (STIs) including HIV, gonorrhoea, chlamydia, trichomoniasis, hepatitis B, genital herpes, syphilis, chancroid, and human papillomavirus infection [12–14]. The situation is particularly challenging for ACB men who account for a disproportionately higher percentage of people living with HIV [15]. The lower prevalence of condom among men is generally attributable to a certain extent to their contraceptive preferences and perception of health risks, and the limiting effect of various sociocultural determinants such as poor health literacy, misconception, care-seeking behaviour, and nonuse of the available services is also well documented [16–19].

The number of ACB immigrants has been increasing constantly in Canada and represents one of the fastest growing ethnic subpopulations in the country. The major driving forces behind this influx are generally the need for safety, better livelihood and income opportunities, better health and living condition, and family reunification [20]. Canada's healthcare system boasts one of the most advanced in the world especially for providing an immigrant-friendly labour market and healthcare landscape. However, a host of challenges continue to persist in providing care in an equitable manner among immigrants from low- and middle-income countries who are more likely to have different health issues and needs compared with the local population [21]. The inherent difficulties in providing care for this disadvantaged population group, coupled with their exposure to risk factors (such as low income and health literacy), can significantly increase their vulnerability to STIs and receiving the health service in a timely manner. Several studies have highlighted this complexity linked to immigrants' sociocultural backgrounds, social capital, educational, employment, and living conditions and legal statuses with their vulnerability for HIV transmission and other sexual health issues [20, 22, 23].

Given this, there is a critical need for expanding the provision and use of condom among ACB men. Unfortunately, lack of quality data and structural gaps in healthcare systems met with financial, socioeconomical, and technological obstacles among ACB men remain a major barrier to solve the situation [24]. To this regard, we undertook the present study with the aim of investigating the factors associated with condom use among ACB men. The data came from the weSpeak project; this represents a five-year multisite (Ottawa, Toronto, London, and Windsor) research programme that aims to (1) assess the sociocultural and sociopolitical conditions that contribute to HIV-related health disparities among ACB men, (2) examine social and behavioural vulnerabilities to HIV among ACB men, including their social identities related to race, class, gender, and sexualities; and (3) generate, appraise, and share new knowledge and support its translation into intervention and practice. weSpeak is a mixed methods study comprising focus groups, in-depth interviews, and a questionnaire survey. The present analysis was based on quantitative data only [25, 26].

## 2. Methods

### 2.1. Sampling and Recruitment

We estimated the survey sample size for each city based on the number of HIV tests performed among heterosexual ACB men over 5 years, 2007–2011, in relation to the total male ACB population aged 15 years and older in the respective cities from the 2011 National Household Survey, with an additional desired level of precision of 5%. For example, 15,465 tests were attributed to ACB men in Toronto, which represents 12% of Toronto's black male population aged 15 and older in 2011. We doubled the initial estimated sample size for Toronto. Moreover, based on the experience with the MaBwana study, [27] we increased the sample size in all four cities by 25% to achieve greater statistical power and also made allowances for questionnaires/cases that were incomplete or ineligible. Our sample estimates were as follows—Toronto: revised sample size estimate (*n* = 324), recruitment target (*n* = 405); Ottawa: initial sample size estimate (*n* = 207), recruitment target (*n* = 259); London: initial sample size estimate (*n* = 160), recruitment target (*n* = 200); and Windsor: initial sample size estimate (*n* = 140), recruitment target (*n* = 175). Based on the targets above, our sample for the survey was close to 1,039 self-identified heterosexual ACB men, which, in the context of research with ACB communities in Canada, constitutes a large sample. The experiences and perspectives of men living with HIV are germane to the study, but ACB men infected with HIV through heterosexual contact represent less than 2% of the adult male ACB population in various cities in Ontario. To support analysis of data on HIV-positive heterosexual men, we recruited at least 167 HIV-positive men (i.e., 167/831 or 20%) consisting of 65 in Toronto, 42 in Ottawa, 32 in London, and 28 in Windsor. This study was limited to participants who mentioned having any sexual partner during the last 12 months.

*Variables*: The outcome variables were knowledge of condom use, attitude towards condom use, and use of condom during the last 12 months. Knowledge was assessed by the question: *are condoms the best way to protect myself against HIV transmission*? The answers were coded as good (if replied yes) and not good (if replied no/don't know). Attitude was assessed as a composite score to a set of questions regarding the use of condom: *is it uncomfortable, is it not appealing, does it make sex unenjoyable, does it enhance sexual pleasure, would avoid if possible, does it mean a sign of concern and responsibility, is it unmanly,* and *just don't like the idea*? Answers for all these items ranged from strongly agree to strongly disagree. Based on the context, some of the answers were reverse recoded to calculate a global score, which was finally divided into quantiles. Those whose answers were in the fourth and fifth quantile were recoded as having positive attitude, and the rest were recoded as “negative attitude.” Condom use during the last 12 months with casual partners was coded as “user” and “nonuser.”

*Explanatory variables:* Literature review was conducted in PUBMED for articles published on similar topics to identify a list of sociodemographic variables that are associated with condom knowledge, attitude, and practice. Based on the results of the review, the following variables were included in the analysis: age (15–19/20–29/30–39 40–49/50–59/60–64/65/+); currently married (No/Yes); ethnicity (Black African/Black Caribbean/Black Canadian/Black American/Black Latin American); education (None/Pre–High School/High School/College/Preuniversity/Completed University); employment (No/Yes); local status (Canadian Citizen/Landed Immigrant & Permanent Resident/Temporary Work Permit/Other); self-reported health (Not Good/Good); no. of partners in last 12 months (1–3/4–5/>5); sexual health visit in last 12 months (No/Yes); and experienced any difficulty in accessing sexual healthcare (Yes/No).

### 2.2. Statistical Analysis

Data were cross-checked by two independent researchers before entering into Stata for analysis. Some of the variables were recoded into smaller categories for the purpose of the regression analysis. Data were analysed by using Stata, version 14, and R. Descriptive analyses were performed to present the basic sociodemographic profile of the participants according to their knowledge, attitude, and use of condom. Logistic regression models were then performed to test the association between outcome and explanatory variables. Three different models were run for three outcome variables. Results of regression analyses were presented as odds ratios and 95% confidence intervals. Statistical significance was set at *p* < 0.05. Level of multicollinearity among the variables used in regression analyses was assessed using variance inflation factors (VIF). The VIF statistic for all three models was below 4, while <10 was indicated the acceptable level of multicollinearity.

## 3. Results

As shown in Table 1, 22.30% of the participants correctly responded that the condom is the best protection against HIV transmission, 31.77% of the men had positive attitude, and 62.43% reported using condom regularly during the last 12 months.

**Figure 1:**
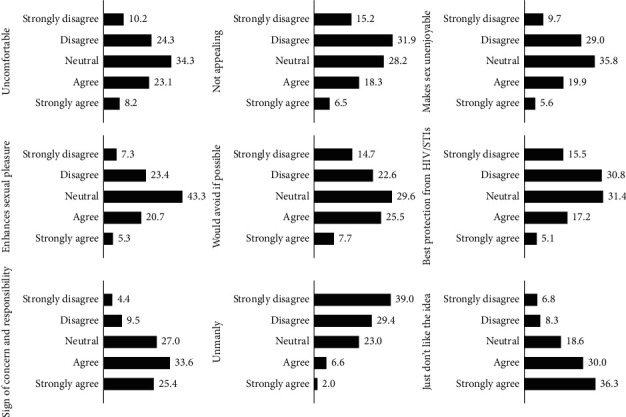
Responses on condom knowledge and attitude.

As shown in Figure 1, more than a quarter of the participants had neutral opinion towards comfort, appealingness, enjoyableness, pleasure-enhancing effect, willing to avoiding, and sign of concern and responsibility regarding condom use.

**Figure 2:**
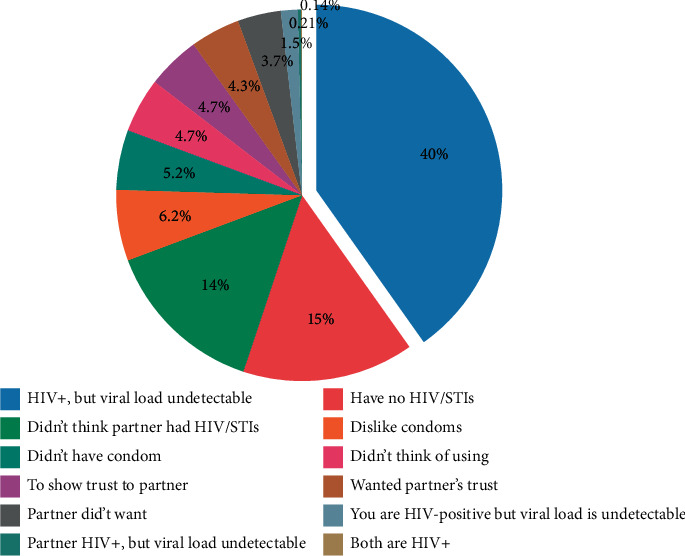
Reasons for not using condoms with the last partner.

Figure 2 shows that the bulk of participants reported undetectable viral load as the main reason for not using condoms. Having no HIV/STIs in them and their partners were the next two most commonly reported reasons for not using condoms.

Results of regression analyses are presented in Table 2. Those in the lower age groups (e.g., 20–29 years) had relative higher odds of having correct knowledge of condom (odds ratio = 1.25, 95% CI = 1.02, 1.53), positive attitude (odds ratio = 2.03, 95% CI = 1.24, 3.34), and use of condom (odds ratio = 1.38, 95% CI = 0.95, 2.02). Those who were currently married had more positive attitude towards condom use compared with their unmarried counterparts (odds ratio = 1.46, 95% CI = 1.20, 1.78). Ethnicity did not show any significant association with any of the three outcomes. Compared with those who had completed university education, those in the secondary education group had lower odds of good knowledge (odds ratio = 0.85, 95% CI = 0.74, 0.98), and those in the college/preuniversity group had lower odds of using condoms during the last 12 months (odds ratio = 0.30, 95% CI = 0.10, 0.94). Having an employment also showed higher odds of having positive attitude towards condom use (odds ratio = 2.19, 95% CI = 1.43, 3.35). Regarding residency status, Canadian residents had higher odds of having correct knowledge of condom (odds ratio = 1.31, 95% CI = 1.11, 1.55) and positive attitude towards condom use (odds ratio = 1.44, 95% CI = 1.09, 1.92). Those who reported being in good physical health had higher odds of having positive attitude towards condom use (odds ratio = 1.58, 95% CI = 1.09, 2.30). Having more than 5 partners during the last 12 months also showed a positive association with having correct knowledge of condom (odds ratio = 1.52, 95% CI = 1.14, 2.02) and positive attitude towards condom use (odds ratio = 1.94, 95% CI = 1.26, 2.97). Having visited sexual health clinics showed a positive association with having correct knowledge of condom (odds ratio = 1.78, 95% CI = 1.30, 2.44), and having experienced difficulty in accessing sexual health showed a negative association towards condom use (odds ratio = 0.45, 95% CI = 0.21, 0.97).

**Figure 3:**
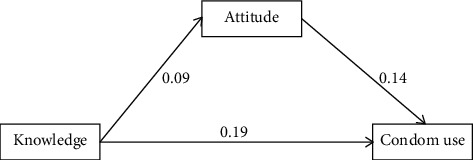
Path analysis showing association between knowledge and attitude towards condom use with its practice.

Figure 3 indicates a weak but positive association between condom knowledge and use (*b* = 0.19). There was also significant mediation of the association between condom knowledge and use by attitude towards condom use. Knowledge of condom use showed a mild correlation (0.14) with attitude and attitude with condom use (0.19).

## 4. Discussion

### 4.1. Main Findings

Based on quantitative data from the weSpeak study, this study aimed to explore the sociodemographic and healthcare-related factors that are associated with knowledge, attitude, and use of condom among African, Caribbean, and black men living in Ontario. Our findings indicate that more than three-quarters of the participants correctly responded that the condom is the best protection against HIV transmission, where less than one-third had positive attitude towards condom use and about two-thirds reported using condoms regularly during the last 12 months. Descriptive analysis also indicated that the bulk of participants reported undetectable viral load and having no HIV/STIs in them and their partners as the main reasons for not using condoms. Contextual data on the nonuse of condoms are critical for addressing the misconceptions and negative attitudes and thereby promoting its use among ACB men.

Among the sociodemographic factors, lower age groups, living with a spouse, having university-level education, Canadian residency, good subjective health, having >5 partners, and having visited sexual health clinics showed a positive association with the outcome variables. However, it is worthy of attention that participants' residency status was found to be an important factor that was contributing to correct knowledge of condom effectiveness in HIV prevention and positive attitude towards condom use. This implies that new comers and nonresidents could be more at risk for HIV and STI transmission, as they may not be up to date with regard to their understanding, their attitude, and condomless sex practice. Surprisingly, ethnic background did not show any significant association with any of the outcomes, signifying that the level of knowledge, attitude, and practice do not vary substantially among the ethnic groups. However, it is worthy of attention that participants' residency status was an important factor for having correct knowledge of condom and positive attitude towards condom use. This is perhaps because the nonresidents or new immigrants are less likely to avail or access the resources that can equip them with proper knowledge and attitude to avoid engaging in risky sexual behaviours [28–32]. As newcomers, especially from low-income settings, people generally face difficulties in getting acquainted with the healthcare system (such as getting health insurance) and making the first move to seeking care. Several factors can be responsible for that: health literacy, official language proficiency, and concept of health communication in a different culture [33–37]. This can be more prominent for sexual and reproductive health issues among men who tend to ignore such issues as temporary and something that will heal itself over time [22, 38–40]. Current sexual health programmes should therefore pay special attention to the individuals with permanent residency status. As expected, having higher education showed a positive association with good knowledge compared with those with the lowest educational level. However, there are no significant associations between education, attitude, and practice. Having an employment and good subjective health also showed higher odds of having positive attitude towards condom use, but not with knowledge and practice.

Interestingly, having more than 5 partners during the last 12 months also showed a positive association with having correct knowledge of condom and positive attitude towards condom use. It is likely that men having multiple relations or more experiences are more likely to peer-learn the correct information and grow protective attitude. At the same, it is important to bear in mind that having multiple partners itself is considered a risky behaviour and not a recommended way of gaining better knowledge and attitude towards condom use. Lastly, having visited sexual health clinics showed a positive association with having correct knowledge of condom, and having experienced difficulty in accessing sexual health showed a negative association towards condom use. Sexual health-seeking behaviour is an important factor for prevention and intervention of HIV and other STIs. Having access to sexual healthcare and care-seeking behaviour are vital for meeting the health and reproductive needs especially among minority and marginalised communities. Results indicated that men who reported having difficulty in accessing care were less likely to have positive attitude towards condom use. This finding is understandable given the fact that an individual's experience and interaction with the healthcare system can greatly influence health-promoting attitude and behaviour. It is therefore recommended that healthcare policymakers take necessary measures to address the negative experiences and difficulties in accessing sexual healthcare among ACB men. Future studies should focus on more healthcare- and culture-related factors to investigate the level of knowledge, attitude, and practice of condom use among racial and marginalised population in Canada.

### 4.2. General Discussion

In Ontario, African, Caribbean, and black men account for almost 60% of the estimated number of HIV-positive people (through heterosexual contact), although they constitute less than 5% of the province's population. However, current HIV research, programming, and policies in Ontario are not aligned with heterosexual ACB men's needs and interests and fail to engage them in community responses to HIV. The weSpeak is a multisite (Ottawa, Toronto, London, and Windsor) project that is targeted to explore the sociocultural and sociopolitical conditions that contribute to HIV-related health disparities among ACB men; examine social and behavioural vulnerabilities to HIV among ACB men, including their social identities related to race, class, gender, and sexualities; generate, appraise, and share new knowledge; and support its translation into intervention and practice. Overall, this project is expected to strengthening multiple-sector partnerships and collaboration in HIV responses of ACB communities; identifying initiatives to strengthen resilience; building critical health literacy; supporting ACB men's involvement in community responses to HIV; building capacity in CBR and policy analysis; and generating new knowledge to reduce HIV-related health disparities in ACB communities to address the HIV epidemic in Ontario and beyond.

### 4.3. Strengths and Limitations of the Study

Findings of the present study highlight the factors associated with knowledge, attitude, and use of condom among ACB men in Ontario, which will contribute to a better understanding of the relevant factors influencing HIV vulnerability among heterosexual ACB men. It may also inform evidence-based policy making in the field. Although the sample population was relatively small, participants were selected cautiously, and the analysis was limited to only sexually active men who reported having a casual partner during the last 12 months. Another important strength was the inclusion of ACB men regarding of their residency status. As we included both Canadian citizens/permanent residents and study/permit holders, it helped get a better comparative picture of the factors that are associated with the outcome variables. We also used path analysis to assess the structural relationship knowledge, attitude, and practice of condom use. More importantly, we included men's medical visit for sexual health clinics in the last 12 months and experience of any difficulty in accessing care, which are good indicators of health-promoting behaviour and are likely to influence both the attitude and practice of condom use. There are some limitations to report as well. We used cross-sectional data for this analysis, which implies that the associations cannot be taken for casual relations. Condom use knowledge refers to the understanding that regular condom utilization is protective against HIV infection, rather than the knowledge of condom using manual. Last but not least, the sample size was not a representative of the general population, and therefore, the findings may not be applicable for the entire male population of Ontario.

## 5. Conclusion

This study was a part of the series of research projects embedded within the weSpeak programme of research. This study investigated the sociodemographic and healthcare-related factors that are associated with knowledge, attitude, and use of condom among heterosexual ACB men in Ontario. In short, more than three-quarters of the participants responded that the condom is the best protection against HIV transmission, less than one-third had positive attitude towards condom use, and about two-thirds reported using condom regularly during the last 12 months. Several sociodemographic and healthcare-related factors were found to be significantly associated with the outcome variables, addressing which could result in better knowledge, attitude, and use of condom in the target population. Although the data were cross-sectional and population was not representative of the province, it is expected that the findings will enhance the current understanding of the potential risk factors of poor condom use and HIV-related knowledge and attitude among ACB men in Ontario. This will aid in designing evidence-based interventions and other initiatives to reduce heterosexual ACB men's vulnerability to HIV and its associated consequences.

## Figures and Tables

**Table 1:** Descriptive analysis of condom knowledge, attitude, and utilization among ACB men in Ontario.

	Knowledge		Attitude		Regular condom user	
	Not good (22.30%)	Good (77.70%)	Not positive (68.23%)	Positive (31.77%)	No (37.57%)	Yes (62.43%)
Age, years						
15–19	5.80	3.41	5.10	2.05	1.47	3.98
20–29	26.81	29.41	28.03	30.14	16.18	36.73
30–39	34.06	33.13	33.44	33.56	34.56	30.97
40–49	20.29	18.58	19.11	19.18	24.26	17.26
50–59	6.52	9.60	8.28	8.90	13.24	7.08
60–64	4.35	3.72	3.50	4.79	5.15	3.54
65/+	2.17	2.17	2.55	1.37	5.15	0.44

Currently married						
No	46.38	43.26	42.77	47.59	20.00	50.67
Yes	53.62	56.74	57.23	52.41	80.00	49.33

Ethnicity						
Black African	67.26	53.55	61.34	48.80	54.92	51.31
Black Caribbean	19.47	23.76	19.33	29.60	24.59	24.08
Black Canadian	10.62	19.86	17.10	17.60	18.03	21.47
Black American	1.77	1.06	0.74	2.40	1.64	1.05
Black Latin American	0.88	1.77	1.49	1.60	0.82	2.09

Education						
None/pre-high school	0.74	1.87	0.65	3.45	2.21	1.33
High school	19.26	12.77	13.87	16.55	12.50	11.56
College/preuniversity	25.19	35.20	30.00	36.55	35.29	34.22
Completed university	54.81	50.16	55.48	43.45	50.00	52.89

Employment						
No	6.36	10.71	10.23	8.00	8.20	7.04
Yes	93.64	89.29	89.77	92.00	91.80	92.96

Local status						
Canadian citizen	49.28	55.73	51.27	58.90	64.71	60.18
Landed immigrant/permanent resident	18.12	14.24	16.24	13.70	12.50	12.39
Temporary work permit (temporary foreign worker)	16.67	13.62	16.24	10.96	9.56	14.16
Other	15.94	16.41	16.24	16.44	13.24	13.27

Self-reported health						
Not good	5.07	4.95	3.82	7.53	5.15	3.98
Good	94.93	95.05	96.18	92.47	94.85	96.02

No. of partners in last 12 months						
1–3	84.21	80.84	85.59	73.95	91.91	75.66
4-5	6.32	9.20	5.51	14.29	2.94	11.50
>5	9.47	9.96	8.90	11.76	5.15	12.83

Sexual health visit in last 12 months						
No	89.22	88.93	88.76	89.47	90.18	88.27
Yes	10.78	11.07	11.24	10.53	9.82	11.73

Experienced any difficulty in accessing						
Yes	24.51	12.50	13.73	20.34	14.29	18.09
No	75.49	87.50	86.27	79.66	88.71	81.91

**Table 2:** Factors associated with condom knowledge, attitude, and use among ACB men in Ontario.

	Knowledge	Attitude	Condom use
Age groups (65/+)			
15–19 years	1.25^*∗*^ [1.02, 1.53]	2.03^*∗∗*^ [1.24, 3.34]	1.38 [0.95, 2.02]
20–29 years	1.15 [0.91, 1.44]	0.89 [0.54, 1.46]	1.58^*∗*^ [1.01, 2.48]
30–39 years	1.78^*∗∗∗*^ [1.41, 2.24]	1.74^*∗*^ [1.12, 2.70]	2.00^*∗∗*^ [1.21, 3.31]
40–49 years	2.68^*∗∗∗*^ [2.12, 3.37]	2.95^*∗∗∗*^ [1.88, 4.64]	2.77^*∗∗∗*^ [1.67, 4.60]
50–59 years	1.38 [0.63, 3.00]	1.24 [0.42, 3.67]	0.12 [0.01, 1.62]
60–64 years	1.20 [0.56, 2.58]	0.91 [0.32, 2.61]	0.09 [0.01, 1.19]

Currently married (no)			
Yes	0.99 [0.82, 1.19]	1.46^*∗∗∗*^ [1.20, 1.78]	1.14 [0.75, 1.74]

Ethnicity (Black African)			
Black Caribbean	1.45 [0.68, 3.09]	1.30 [0.46, 3.71]	0.22 [0.02, 2.94]
Black Canadian	1.31 [0.62, 2.81]	1.60 [0.57, 4.54]	0.09 [0.01, 1.19]
Black Latin American	1.23 [0.57, 2.64]	1.05 [0.36, 3.04]	0.14 [0.01, 1.86]

Education (completed university)			
College/Preuniversity	0.77 [0.47, 1.26]	0.60 [0.27, 1.31]	0.30^*∗*^ [0.10, 0.94]
High school	0.85^*∗*^ [0.74, 0.98]	0.86 [0.68, 1.10]	0.89 [0.66, 1.21]
None/pre-high school	1.17 [0.92, 1.49]	1.09 [0.72, 1.65]	0.86 [0.44, 1.64]

Employment (no)			
Yes	0.97 [0.79, 1.19]	2.19^*∗∗∗*^ [1.43, 3.35]	1.06 [0.71, 1.56]

Residency status (other)			
Canadian citizen	1.31^*∗∗*^ [1.11, 1.55]	1.44^*∗*^ [1.09, 1.92]	1.13 [0.76, 1.68]
Landed immigrant or permanent resident	1.21 [0.99, 1.48]	1.34 [0.95, 1.90]	1.26 [0.79, 2.01]

Self-reported health (not good)			
Good	1.33 [1.00, 1.77]	1.58^*∗*^ [1.09, 2.30]	0.37 [0.08, 1.70]

No. of partners in last 12 months (1–3)			
4-5	1.27 [0.95, 1.68]	1.18 [0.80, 1.75]	1.20 [0.32, 4.45]
>5	1.52^*∗∗*^ [1.14, 2.02]	1.94^*∗∗*^ [1.26, 2.97]	0.94 [0.27, 3.30]

Visited sexual health clinic (no)			
Yes	1.78^*∗∗∗*^ [1.30, 2.44]	1.15 [0.65, 2.00]	1.70 [0.48, 6.01]

Difficulty in getting sexual healthcare (no)			
Yes	0.89 [0.55, 1.43]	0.45^*∗*^ [0.21, 0.97]	1.24 [0.41, 3.77]

N.B.: ^*∗*^ = significant at *p* < 0.05. ^*∗∗*^ = significant at *p* < 0.01. ^*∗∗∗*^ = significant at *p* < 0.001.

## Data Availability

Data can be made available upon request to the corresponding author.
